# Spontaneous Pyometra in a Very Elderly Woman Revealing Serous Endometrial Carcinoma: A Case Report

**DOI:** 10.7759/cureus.86137

**Published:** 2025-06-16

**Authors:** Stefanos Flindris, Chrysoula Margioula-Siarkou, Georgia Margioula-Siarkou, Konstantinos Dinas, Stamatios Petousis

**Affiliations:** 1 2nd Department of Obstetrics and Gynaecology, Aristotle University School of Medicine, Thessaloniki, GRC

**Keywords:** elderly woman, hysterectomy, postmenopausal infection, pyometra, serous endometrial carcinoma

## Abstract

Pyometra is a rare accumulation of purulent material within the uterine cavity that most often arises in postmenopausal women with cervical stenosis. We report the case of an 86-year-old multiparous female patient with a history of multiple dilatation and curettage procedures who presented with a 15-day history of lower abdominal pain, spotting, and systemic signs of infection. Transvaginal ultrasound and contrast-enhanced CT confirmed a distended uterus filled with hypoechogenic fluid without evidence of perforation. Initial management with drainage under antibiotic coverage was complicated by paralytic ileus and rising inflammatory markers, prompting an emergency laparotomy. A total hysterectomy with bilateral salpingo-oophorectomy, appendectomy, and infracolic omentectomy was performed. Histopathology revealed a serous endometrial adenocarcinoma (International Federation of Gynecology and Obstetrics or FIGO stage IIC) confined to an endometrial polyp. Postoperatively, the patient recovered uneventfully, proceeded to adjuvant radiotherapy and brachytherapy because of occult malignancy, and remained disease-free at six months of follow-up. This case underscores the importance of early recognition and definitive surgical management of pyometra in elderly patients, as well as careful histopathologic evaluation to exclude underlying malignancy.

## Introduction

Pyometra is an uncommon but clinically important condition in which the uterine cavity fills with purulent material, distinguishing it from endometritis. Its reported incidence ranges from 0.01 to 0.5% among gynecologic patients, which rises to 13.6% in older adults [1]. The most frequent underlying factor is narrowing of the cervical canal, often resulting from postmenopausal tissue thinning, prior radiation treatment, surgical procedures, or congenital anomalies such as an imperforate hymen. Other contributing factors include pelvic inflammatory disease, the use of an intrauterine device, and changes after childbirth [2]. However, malignancy is a less common cause; it is more likely to coexist with pyometra in elderly women [3].

Clinically, patients may present with bleeding after menopause, abnormal vaginal discharge, fever, and lower abdominal pain; however, some remain without symptoms until complications develop. Serious outcomes can include sepsis, rupture of the uterus, and inflammation of the abdominal lining, all of which carry a significant risk of death. Specifically, a review conducted in 2012 reported only 81 cases of uterine rupture between 1949 and 2011, with a 25% mortality rate [4], while the mortality rate reaches 40% in patients with spontaneous perforation of pyometra [5].

In addition, prompt diagnosis depends on maintaining a high index of suspicion. Ultrasound, CT, and MRI are all effective for detecting fluid within the uterus and assessing the integrity of the uterine wall. In patients whose cervical canal is narrowed by atrophy, draining the purulent material with dilation techniques may be difficult and increases the risk of perforation during hysteroscopy or curettage [1,3]. Consequently, antibiotic therapy should be tailored to the likely pathogens, although standard regimens may be less effective when the drainage is incomplete. Despite that, the condition may recur in a few weeks or months after conservative treatment [6].

Given these challenges and the potential for inadequate treatment, surgery is often the preferred option. Definitive management typically involves removal of the uterus, and both the ovaries and fallopian tubes. This strategy not only ensures complete evacuation of the infected material and relief of obstruction, but also provides tissue for histological examination, thereby excluding occult malignancy, and minimizes the likelihood of recurrence and severe complications [7].

Importantly, while malignancy is an uncommon cause of pyometra, it must always be considered, especially in elderly women, as it can remain clinically silent and only be revealed upon surgical exploration and histopathologic evaluation. Here, we describe the case of an 86-year-old female patient who presented with pyometra more than three decades after her last menstrual period, in whom definitive surgery revealed a serous endometrial carcinoma.

## Case presentation

An 86-year-old female patient, Gravida 2, Para 2, Abortus 0 (G2P2A0 or the patient had been pregnant twice, had two deliveries after 20 weeks of gestation, and no abortions or miscarriages before 20 weeks of gestation), with a history of normal labor, presented to the emergency department with a 15-day history of progressively worsening hypogastric pain, associated with spotting type of vaginal bleeding and diarrhea. The patient had a significant medical history, including glaucoma, hypertension, and a cholecystectomy seven years ago. She denied any allergies or smoking history.

Fifty years earlier, the patient had undergone multiple dilatation and curettage (D&C) procedures due to abnormal uterine bleeding. Despite these interventions, she had a normal postmenopausal course until the onset of her current symptoms. She had been in menopause since the age of 51 years.

Upon initial evaluation by a private gynecologist, an enlarged uterus with a hypoechogenic fluid accumulation was noted on ultrasound. Due to cervical stenosis, attempts to obtain a biopsy and perform drainage via a pipelle were unsuccessful. The gynecologist recommended further examination with an MRI, but the patient did not return for follow-up until her symptoms worsened.

After seven days of the initial consultation, the patient presented to the emergency department with intolerable lower abdominal pain, fever (37.6°C), and chills. Her pulse was 85 beats per minute, regular in rhythm and rate, respiratory rate was 16 breaths per minute, and blood pressure was 135/90 mmHg. The central nervous system, cardiovascular system, and respiratory system examination findings were found to be normal. On physical examination, the uterus was noted to be enlarged and was palpable in the lower abdomen, and extreme tenderness was elicited upon bimanual examination, including cervical motion tenderness. A vaginal examination revealed an atrophic, firm cervix that was level with the vaginal walls. The abdominal mass was palpated as being continuous with the cervix, with cervical movements being transmitted to the mass, suggesting that it originated from the uterus. Laboratory findings revealed a white blood cell (WBC) count of 36,000/μL, with 95% neutrophils, a C-reactive protein (CRP) of 266 mg/L, and mild elevation of D-dimers. Subsequent laboratory values during her hospital course are summarized in Table 1.

**Table 1:** Laboratory values with reference ranges WBC: White blood cells; CRP: C-reactive protein; FEU: Fibrinogen equivalent units

Parameter	Result	Reference range
WBC (initial), 10^3^/µL	36	4–11
Neutrophils (initial), %	95	40–70
CRP (initial), mg/L	266	<5
D-Dimer (initial), mg/mL FEU	Mildly elevated	<500
WBC (12 h post-drainage), 10^3^/µL	31	4–11
Neutrophils (12 h post-drainage), %	95	40–70
CRP (12 h post-drainage), mg/L	280	<5
WBC (pre-surgery), 10^3^/µL	47	4–11
Neutrophils (pre-surgery), %	97	40–70
CRP (pre-surgery), mg/L	320	<5
WBC (discharge day), 10^3^/µL	8.5	4–11
Neutrophils (discharge day), %	60	40–70
CRP (discharge day), mg/L	8	<5

**Figure 1:**
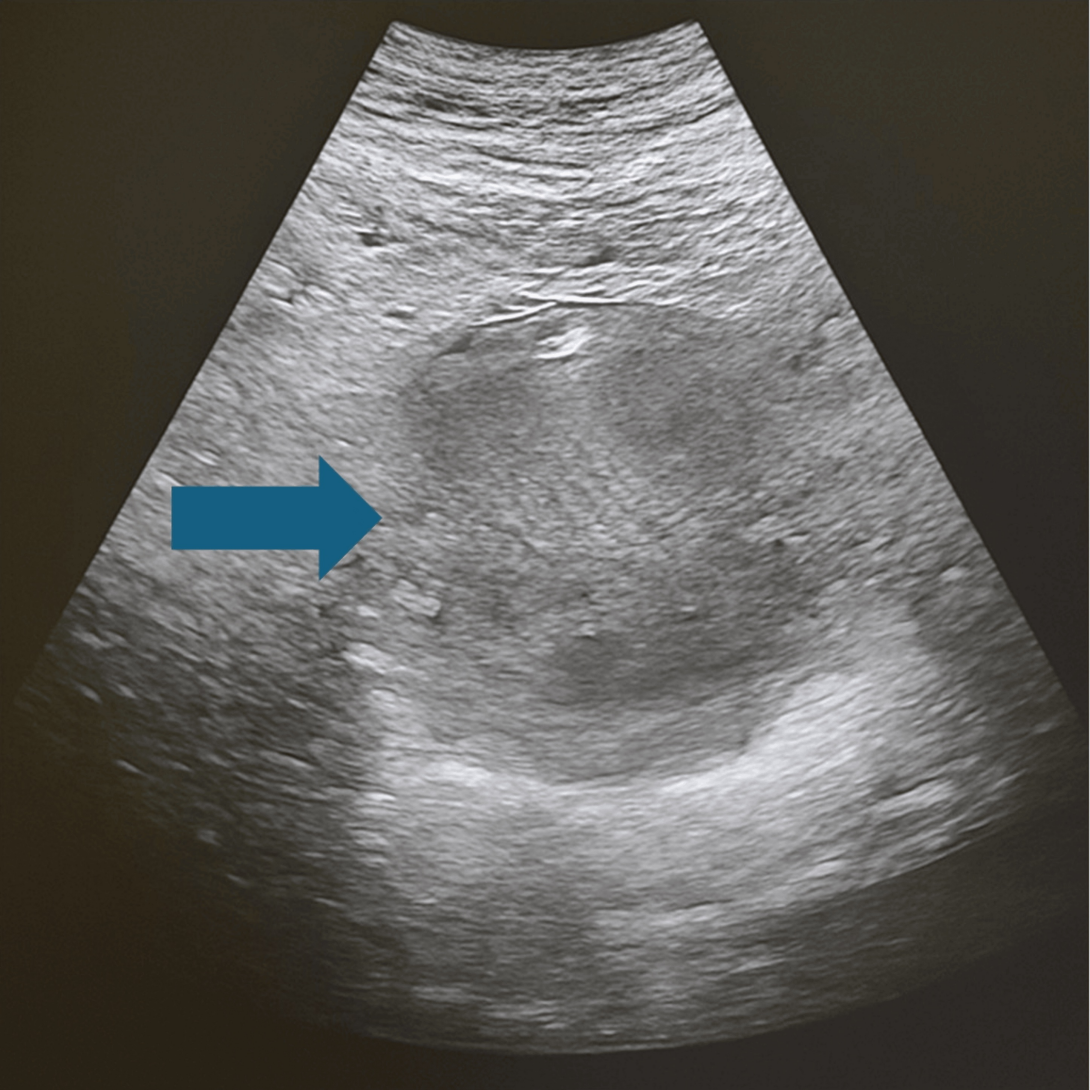
Ultrasound image demonstrating a markedly enlarged uterus with a hypoechogenic intrauterine fluid collection consistent with pyometra (arrow) No evidence of free peritoneal fluid was observed, arguing against uterine perforation or associated peritonitis.

A transvaginal ultrasound confirmed a large uterus with hypoechogenic fluid accumulation with no free peritoneal fluid indicating perforation and septic peritonitis (Figure 1).

**Figure 2:**
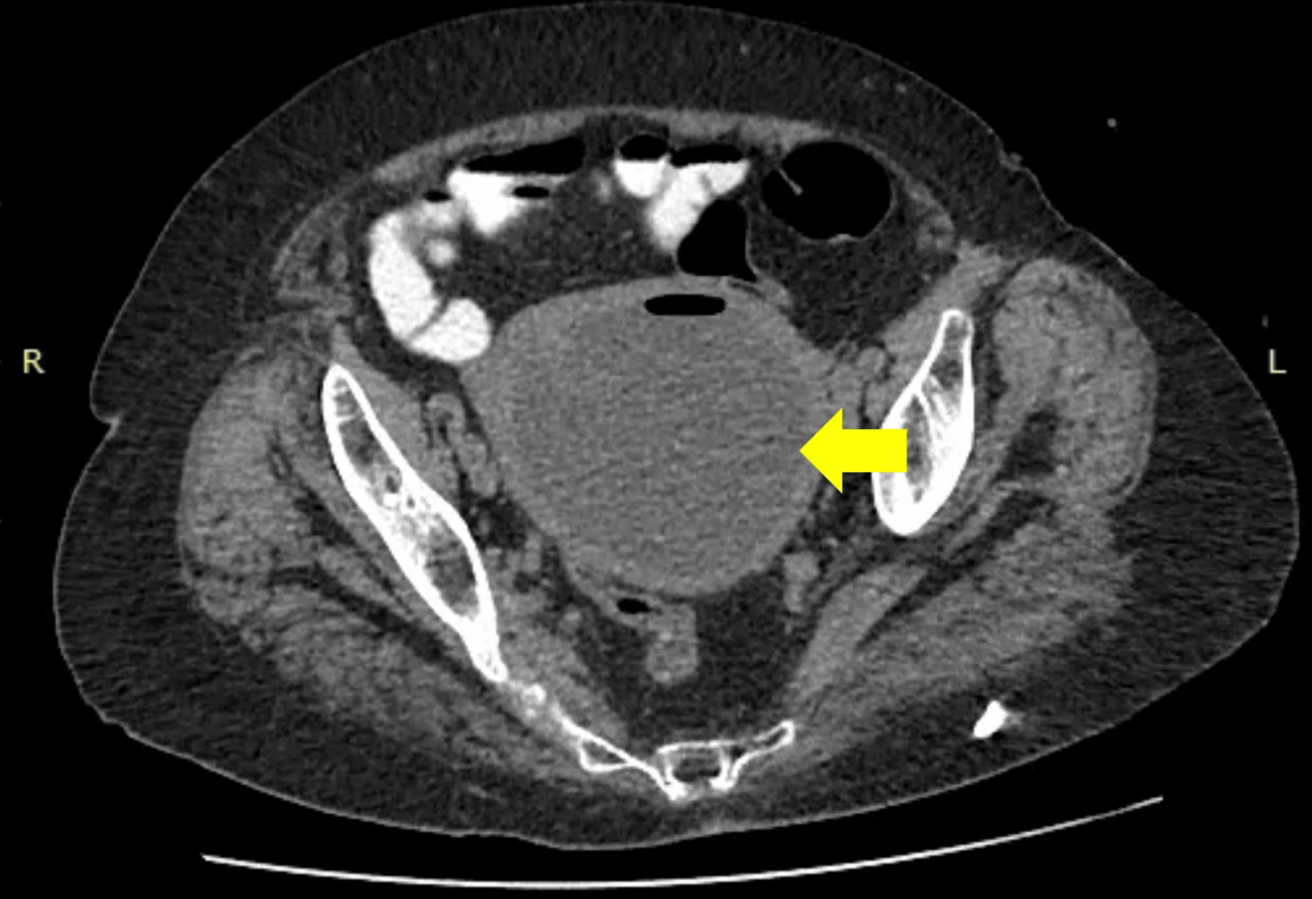
Contrast-enhanced abdominal CT scan showing a distended uterus filled with low-attenuation fluid, consistent with pyometra (yellow arrow) No signs of uterine rupture or free intraperitoneal air or fluid were identified, supporting a diagnosis of unperforated pyometra.

The clinical findings were suggestive of pyometra, prompting a surgical evaluation. After a multidisciplinary discussion with the gynecologists, we decided to perform a contrast-enhanced abdominal CT scan (Figure 2).

Axial CT through the pelvis demonstrated a markedly distended uterine cavity filled with homogeneous low-attenuation fluid, producing an appearance of classic fluid collection. The endometrial lining was thinned, and peripheral enhancement of the myometrium could be seen. A small fluid-air level was present, consistent with superimposed infection. The uterine contour was smooth and there was no evidence of adjacent abscess or free intraperitoneal fluid. These findings were consistent with pyometra, leading to the patient's hospitalization for further management.

Empiric antibiotic therapy with piperacillin sodium-tazobactam and metronidazole was initiated. After 12 hours from the initial admission an attempt to drain the purulent component of the endometrium transvaginally, using an endometrial Pipelle (Endosampler, MedGyn Products, Illinois, USA) and Foley catheter (Wellcare Latex foley catheter, Well Lead Medical Co. Ltd, Guangzhou, China) was successful, with foul-smelling purulent material drained from the endometrial cavity. Samples were sent for cytological, histopathological, and microbiological analysis.

The patient experienced severe pain and discomfort following the drainage procedure. Two hours after the intervention, the WBC count rose to 31,000/μL, with 95% neutrophils, and CRP increased to 280 mg/L. The patient complained about absence of gas and feces (symptoms of ileus) for three days before admission to the hospital and the clinical examination revealed limited bowel sounds. An abdominal X-ray with gastrographin showed no obvious bowel obstruction but confirmed paralytic ileus due to significant uterine inflammation. Clinical deterioration was observed, including worsening leukocytosis at 47,000/μL with 97% neutrophils and rising CRP levels (320 mg/L), indicating sepsis. Hence, we decided to conduct an emergency laparotomy. The patient was taken to the operating room for urgent surgical intervention.

**Figure 3:**
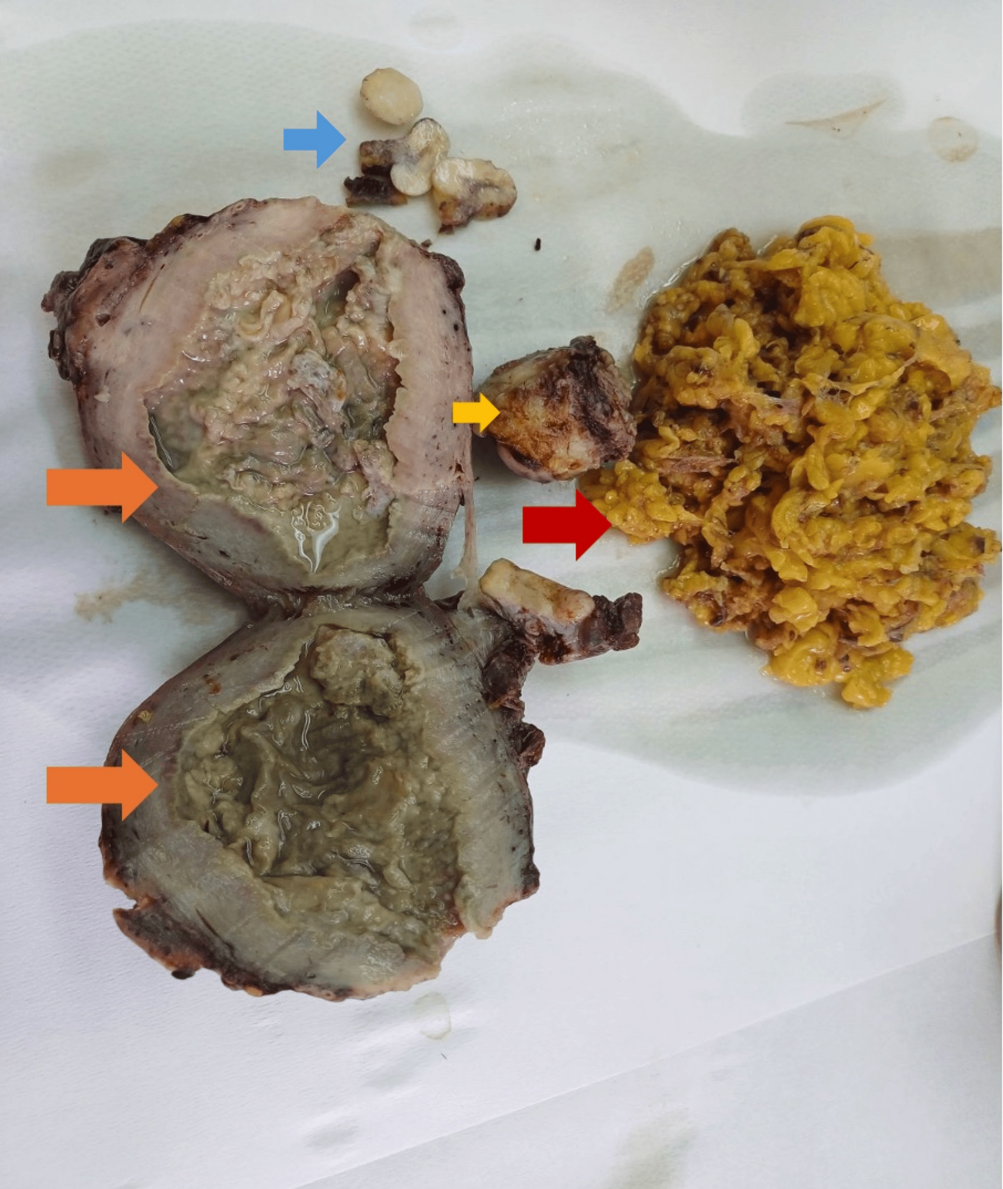
Gross surgical specimens following total hysterectomy and infracolic omentectomy The uterine corpus has been bisected to reveal a markedly distended endometrial cavity filled with purulent material (orange arrows), consistent with advanced pyometra. The cervix is indicated by the yellow arrow. The blue arrow points to the endometrial polyp in which serous endometrial carcinoma was identified on histologic evaluation. The resected infracolic omentum appears as lobulated yellow tissue to the right (red arrow). Appendix and adnexal tissues, although removed, are not shown.

A midline vertical laparotomy was performed, revealing a massive uterus, approximately the size of a 16-week gestation, with a very fragile consistency and foul odor. Due to the advanced stage of the infection and the poor prognosis of preserving the uterus, a total hysterectomy with bilateral oophorectomy, appendectomy, and infracolic omentectomy were performed. Adnexal bilaterally and infracolic omentum were resected to exclude concurrent pathology and ensure oncologic safety. Surgical specimens are depicted in Figure 3.

A single dose of amicasin 500 mg was administered intraoperatively. Abundant normal saline lavage was used to irrigate the peritoneal cavity, and careful hemostasis was achieved by removing the inflammatory tissue from the left lateral parametrium and reassuring the ureter’s integrity, and a drain was placed in the pouch of Douglas.

The patient was transferred to the intensive care unit (ICU) for stabilization and monitoring for 24 hours postoperatively. During this period, she was managed with meropenem and tigecycline, along with thromboprophylaxis using tinzaparin 6000 IU once a day and graduated compression stockings. The patient's postoperative course was uneventful, with gradual resolution of her inflammatory markers. The microbial cultures of the endometrial cavity identified Escherichia coli and Streptococcus. Furthermore, Escherichia coli was also detected in urine cultures. On the first postoperative day, the patient was able to pass gas and stool. The drain was removed on the third postoperative day, and she was discharged on the fifth postoperative day, completely recovered and afebrile. Cefazolin and metronidazole pos were prescribed to the patient for seven days, as well as tinzaparin 6000 IU once a day for 30 days.

The pathology report identified a serous endometrial adenocarcinoma measuring 0.4 cm in maximum diameter, arising within a polyp at the uterine fundus. The tumor was classified as International Federation of Gynecology and Obstetrics (FIGO) stage IIC. Immunohistochemical staining showed strong (>90%) p53 expression, diffuse positivity for p16 and vimentin, and focal positivity for estrogen receptors.

Following a multidisciplinary review by our Gynecologic Oncology Unit, and given the tumor's confinement within a polyp, the presence of only incipient myometrial invasion, and the patient's advanced age (86 years) and frailty, it was decided to proceed with 30 sessions of external beam radiotherapy (EBRT) and three sessions of brachytherapy. Adjuvant chemotherapy was omitted, as the risk of treatment-related toxicity in this patient population may outweigh the potential oncologic benefit. This decision aligns with international guidelines (National Comprehensive Cancer Network (NCCN), European Society for Medical Oncology, European Society of Gynaecological Oncology, and European Society for Radiotherapy and Oncology (ESMO-ESGO-ESTRO)), which recommend that systemic therapy be carefully individualized in elderly or frail patients, particularly when the tumor burden is limited [8,9]. At the six-month postoperative follow-up, the patient remained in excellent clinical condition, with no evidence of recurrence on clinical or radiological assessment.

## Discussion

Pyometra is a rare but serious condition characterized by the accumulation of purulent material within the uterine cavity, typically resulting from cervical stenosis, which obstructs the outflow of uterine secretions [7]. While cervical stenosis due to a malignancy is a well-established cause, senile endometritis associated with cervical stenosis, as seen in this case, is also a significant contributor, particularly in elderly, postmenopausal women [1,3].

Pyometra can result from several etiologies, including cervical and endometrial malignancies, prior radiation therapy, benign conditions such as endometrial polyps and senile endometritis (pyometra), idiopathic cervical canal stenosis, and postoperative infections [10]. Epidemiological data suggest that malignancies account for 22.2% of pyometra cases, genital tract abnormalities for 3.7%, and idiopathic causes for the majority (up to 74.1%) of cases [1]. The infection is caused by various microorganisms, most commonly Escherichia coli, Bacteroides spp., Streptococcus, and other anaerobes [11]. In the present case, pus cultures revealed Gram-negative rods on microscopy, but no bacterial growth was detected in culture, possibly due to prior antibiotic exposure or limitations in culture sensitivity.

The classic clinical triad of pyometra includes lower abdominal pain, postmenopausal bleeding, and purulent vaginal discharge. This may be accompanied by systemic symptoms such as fever with chills, nausea, vomiting, diarrhea, and generalized weakness [1,5]. However, diagnosis can be challenging, particularly in elderly patients or those with a history of cervical procedures, where symptoms may be subtle or atypical [12]. This highlights the importance of a high index of suspicion, thorough clinical evaluation, and appropriate imaging for diagnosis.

In the current case, the patient’s history of multiple dilatation and curettage (D&C) procedures likely contributed to cervical stenosis and subsequent accumulation of pus. If left untreated, pyometra can result in life-threatening complications such as peritonitis, sepsis, hemorrhagic shock, and spontaneous uterine rupture [2,13]. The typical age of ruptured pyometra is reported to be 73.8 years, with a mortality rate ranging between 25% and 40% [4,10].

Initial management of pyometra includes the prompt initiation of broad-spectrum intravenous antibiotics targeting aerobic and anaerobic flora. Surgical drainage, typically via cervical D&C, is often necessary [11]. However, in patients with a thinned uterine wall or suspected perforation, this procedure must be performed with caution to avoid iatrogenic rupture [7,11]. Recurrence is not uncommon and has been reported two to 11 months following conservative treatment [6]. In select cases, particularly where there is uterine perforation, ongoing sepsis, or extensive tissue destruction, hysterectomy may be required. The decision to perform hysterectomy should be individualized based on the patient's comorbidities, infection severity, reproductive status, and risk of recurrence [4,13].

Prognosis largely depends on the underlying cause. Patients with pyometra unrelated to malignancy generally have a more favorable outcome compared to those with cancer-related disease [6]. Therefore, histopathological assessment of endometrial tissue is crucial to exclude underlying malignancy [4]. Additionally, pus samples should be sent for culture and sensitivity testing to guide antimicrobial therapy. In regions with high tuberculosis prevalence, Mycobacterium tuberculosis polymerase chain reaction (PCR) testing or culture should be considered [14].

Imaging plays a vital role in diagnosis and management. Transvaginal ultrasound (TVU) is the first-line investigation and allows for the assessment of uterine distension and endometrial fluid [13,14]. If the diagnosis remains uncertain, or complications such as rupture are suspected, contrast-enhanced CT or MRI can delineate the extent of infection, identify uterine wall integrity, and detect any involvement of adjacent pelvic structures. These modalities are particularly helpful in identifying pyoperitoneum or uterine rupture, which may require emergent surgical intervention [1,12].

International guidelines from the ESMO-ESGO-ESTRO consensus recommend multimodal adjuvant treatment, including both chemotherapy and radiotherapy for high-risk uterine serous carcinoma, reflecting its aggressive behavior and high propensity for extrauterine spread, even in early stages [15]. In the Gynecologic Oncology Group protocol 249 (GOG-249) trial, patients with high-intermediate or high-risk early-stage disease who received only pelvic EBRT had similar recurrence-free and overall survival rates compared to those treated with vaginal brachytherapy plus three cycles of carboplatin/paclitaxel, and experienced fewer acute side effects, supporting a radiotherapy-only approach for appropriately selected patients such as our patient [16]. A recent multi-institutional study of early-stage uterine serous carcinoma confirmed that tailoring adjuvant therapy to individual patient and tumor characteristics can achieve excellent outcomes [17]. In elderly or frail patients, comprehensive geriatric assessment is essential to guide treatment intensity. Dose-reduced or single-agent regimens (e.g., carboplatin monotherapy) may preserve quality of life without compromising efficacy, as outlined in practical geriatric oncology frameworks [18].

## Conclusions

In conclusion, pyometra remains a rare yet potentially life-threatening condition, particularly in elderly women, where delayed diagnosis may result in catastrophic complications such as uterine rupture, peritonitis, and septic shock. This case underscores the importance of maintaining a high index of suspicion in postmenopausal patients presenting with nonspecific abdominal symptoms, especially in those with a history of cervical procedures or atrophic changes. The combination of targeted imaging, early empirical antibiotic therapy, and timely surgical intervention is critical to improving outcomes. Notably, this case revealed an underlying serous endometrial carcinoma, an uncommon but significant finding that highlights the necessity of careful histopathologic evaluation. A multidisciplinary approach remains essential for diagnosis, management, and oncologic decision-making. Prompt recognition and definitive treatment can not only resolve the infection but also uncover occult malignancies, ultimately altering the clinical trajectory and prognosis of this vulnerable population.
